# Switchable and Dual-Tunable Multilayered Terahertz Absorber Based on Patterned Graphene and Vanadium Dioxide

**DOI:** 10.3390/mi12060619

**Published:** 2021-05-27

**Authors:** Hongyao Liu, Panpan Wang, Jiali Wu, Xin Yan, Xueguang Yuan, Yangan Zhang, Xia Zhang

**Affiliations:** 1 State Key Laboratory of Information Photonics and Optical Communications, Beijing University of Posts and Telecommunications, Beijing 100876, China; liuhongyao1230@gmail.com (H.L.); panpanono@163.com (P.W.); Flowing_Kinga@bupt.edu.cn (J.W.); yuanxg@bupt.edu.cn (X.Y.); zhang@bupt.edu.cn (Y.Z.); xzhang@bupt.edu.cn (X.Z.); 2 School of Information and Communication Engineering, Beijing University of Posts and Telecommunications, Beijing 100876, China

**Keywords:** multilayered, switchable, dual-tunable, terahertz, patterned graphene, phase-change material vanadium dioxide

## Abstract

In this paper, a switchable and dual-tunable terahertz absorber based on patterned graphene and vanadium dioxide is proposed and analyzed. By controlling the Fermi level of graphene and the temperature of vanadium dioxide, the device’s function can be switched and its absorbing properties can be tuned. When the vanadium dioxide is in an insulator state, the device can be switched from near-total reflection (>97%) to ultra-broadband absorption (4.5–10.61 THz) as the Fermi level of graphene changes from 0 to 0.8 eV. When the vanadium dioxide is changed to a metal state, the device can act as a single-band absorber (when the Fermi level of graphene is 0 eV) and a dual-band absorber with peaks of 4.16 THz and 7.3 THz (when the Fermi level of graphene is 0.8 eV). Additionally, the absorber is polarization-insensitive and can maintain a stable high-absorption performance within a 55° incidence angle. The multilayered structure shows great potential for switchable and tunable high-performance terahertz devices.

## 1. Introduction

The efficient absorption of incident waves in terahertz (THz) bands is of significance for a wide range of applications such as imaging, food quality control, communication, spectroscopy, and sensing devices [1,2,3,4,5,6]. Metamaterial THz technology has been widely used and investigated in absorbers [7,8,9,10,11]. Graphene is considered one of the most promising metamaterials for tunable THz absorbers since its conductivity can be easily changed either by manipulating the Fermi energy through chemical doping or by applying a gate voltage [12,13,14,15]. Owing to its distinctive electromagnetic and optical properties, graphene is widely considered one of the most effective and promising metamaterials for designing tunable THz absorbers. Different broadband absorbers made of graphene with patterned disks and ribbons have been widely studied [7,16,17,18,19]. Multilayer structure graphene-based absorbers and dual-band absorbers have also been proposed and analyzed [1,20,21,22,23,24]. However, the tunability of graphene absorbers is still limited since, in practice, the Fermi energy level can only be tuned within a small range of 0–0.9 eV [25,26]. Moreover, most of the absorbers exhibit a single and unswitchable function that cannot meet various applications.

An efficient approach for realizing switchable and tunable terahertz absorbers is to combine other materials with graphene. This achieves switchable functionality and provides extra tunability. Vanadium dioxide (VO_2_) is an important phase change material that represents an insulator-to-metal transition. Its conductivity can be increased by four to five orders of magnitude under light, thermal approaches, and external stimuli [27,28]. When its temperature exceeds the temperature required for phase transition (68 °C), a sharp change in conductivity occurs until a conductivity of 2 × 10^5^ S/m is attained. This phase change leads to a reversible transition of infrared light from transmission to reflection [29,30]. VO_2_ can undergo remarkable changes in its electromagnetic characteristics and can also be applied in tunable broadband THz absorber designs [31,32]. In this work, we designed a multilayered THz absorber based on the combination of patterned graphene and VO_2_. By controlling the Fermi levels of graphene and the temperature of VO_2_, the device’s function can be switched and its absorbing properties can be tuned. When VO_2_ is in an insulator state, the device can be switched from near-total reflection (>97%) to ultra-broadband absorption 6.11 THz (4.5–10.61 THz) as the Fermi level of graphene changes from 0 to 0.8 eV. When VO_2_ is transmitted to a metallic state, the device can act as a single-band absorber with an absorption band of 0.84 THz (when the Fermi energy level of graphene is 0 eV) and a dual-band absorber with absorption peaks of 4.16 THz and 7.3 THz (when the Fermi energy level of graphene is 0.8 eV). Moreover, the absorber is polarization-insensitive and can maintain a stable high-absorption performance within a 60° incidence angle. In this work, the absorption mechanism is analyzed first with an impedance matching concept [33] and then investigated by electric field distribution. Our results indicate that the device shows great potential for switchable and tunable terahertz applications.

## 2. Materials and Model

### 2.1. Patterned Graphene

As a 2D material, the surface impedance of graphene is characterized by Kubo’s formula [34]:

(1) $$\begin{array}{c} \sigma_{g}(f,\mu_{c},\tau ,Τ)=\frac{je^{2}(2\pi f-j\tau^{-1})\hbar }{\pi \hbar^{2}}[\frac{1}{{\left(2\pi f-j\tau^{-1}\right)}^{2}}\int_{0}^{\infty }\varepsilon \left(\frac{\partial f_{d}\left(\varepsilon \right)}{\partial \varepsilon }-\frac{\partial f_{d}\left(-\varepsilon \right)}{\partial \varepsilon }\right)d\varepsilon \\ -\int_{0}^{\infty }\frac{f_{d}\left(-\varepsilon \right)-f_{d}\left(\varepsilon \right)}{{\left(2\pi f-j\tau^{-1}\right)}^{2}-4{\left(\varepsilon /\hbar \right)}^{2}}d\varepsilon \end{array}$$

where $f_{d}\left(\varepsilon \right)={\left[\exp\left(\varepsilon -\frac{\mu_{c}}{K_{B}T}\right)+1\right]}^{-1}$ is the Fermi–Dirac distribution, and $\mu_{c}$ and τ are the chemical potential and the relaxation time of graphene, respectively. ℏ, $K_{B}$, and T are the reduced Planck constant, the Boltzmann constant, and the temperature in Kelvin, respectively. In addition, the surface conductivity of graphene can be divided into two parts: $\sigma_{g}=\sigma_{\mathrm{intra}}+\sigma_{\mathrm{inter}}$, where $\sigma_{\mathrm{inter}}$ is the inter-band conductivity and $\sigma_{\mathrm{intra}}$ is the intra-band conductivity. Compared with $\sigma_{\mathrm{intra}}$, the inter-band conductivity ($\sigma_{\mathrm{inter}}$) in the terahertz band can be safely neglected based on the Pauli exclusion principle. Finally, the surface conductivity of graphene can be approximated to be a Drude model [35]:

(2) $$\sigma_{g}(f,\mu_{c},\tau ,Τ)\approx \sigma_{\mathrm{int}\mathrm{ra}}(f,\mu_{c},\tau ,Τ)=\frac{e^{2}\mu_{c}}{\pi \hbar^{2}}\frac{j}{\left(2\pi f+j\tau^{-1}\right)}$$

The surface conductivity of graphene can change by controlling its chemical potential. The relation of $\mu_{c}$ and bias voltage ($V_{g}$) can be expressed by the following approximate form [36]:

(3) $$\mu_{c}=\hbar v_{F}\sqrt{\frac{\pi \varepsilon_{0}\varepsilon_{h}v_{g}}{eh}}$$

where $\varepsilon_{h}$ is the permittivity of the dielectric layer,$\;\varepsilon_{0}$ is the permittivity of the vacuum, $v_{g}$ is the bias voltage, e is the electron charge, and $v_{F}$ is the Fermi velocity. The surface impedance ($Z_{g}$) of the graphene can be written as follows [37]:

(4) $$Z_{g}=\frac{1}{\sigma_{g}}$$

**Figure 1:**
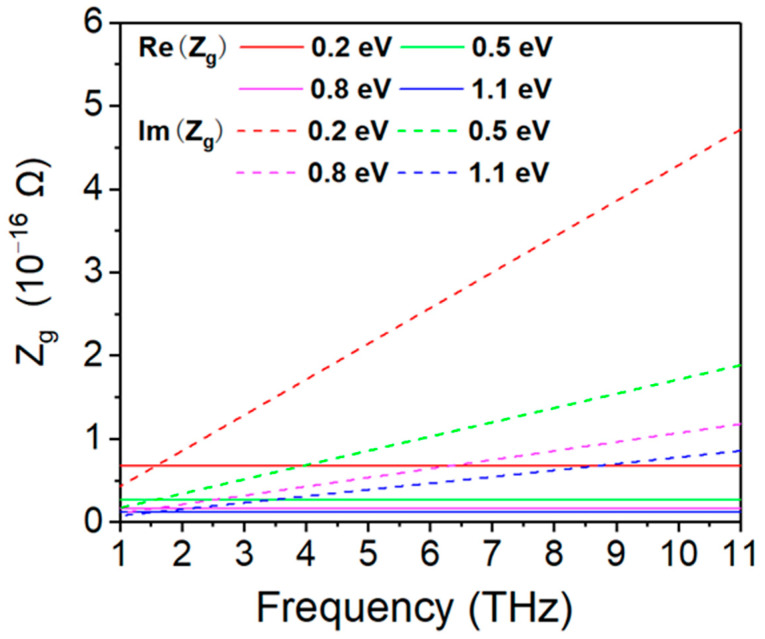
Surface impedance versus frequency of patterned graphene.

In our simulations, we assumed a relaxation time of τ=0.1 ps and a temperature of T=300 K. The finite element method was used to investigate the absorption performance of the proposed absorber in the frequency range of 1 to 11 THz. We numerically calculated the real and imaginary parts of the surface impedance of the graphene film with different chemical energies. The results are shown in Figure 1. The real part remains constant with an increase in the frequency, whereas the imaginary part increases monotonously as the frequency increases. It can also be observed that the real and imaginary parts of the surface impedance decrease as the chemical potential of graphene increases. Tuning can be achieved by controlling the gate voltage using an ion-gel top configuration [38]. This changes the surface impedance of the patterned graphene. Therefore, a tunable absorber is achievable.

### 2.2. Phase-Change Material VO_2_

Within THz range, the relative permittivity of VO_2_ can be described by the Drude model [39]:

(5) $$\varepsilon_{{\mathrm{VO}}_{2}}(\omega )=\varepsilon_{\infty }-\frac{\omega_{P}^{2}(\sigma_{{\mathrm{VO}}_{2}})}{\omega^{2}+i\gamma \omega }$$

(6) $$\omega_{P}^{2}(\sigma_{{\mathrm{VO}}_{2}})=\frac{\sigma_{{\mathrm{VO}}_{2}}}{\sigma_{0}}\omega_{P}^{2}(\sigma_{0})$$

where $\varepsilon_{\infty }$ is the infinite frequency permittivity with a numerical value of 12, $\gamma =5.75\times 10^{13}$ s^−1^ expresses the damping frequency, and ω is the angular frequency of the incident wave. Additionally, $\sigma_{0}=3\times 10^{5}$ S/m and $\omega_{p}(\sigma_{0})=1.4\times 10^{15}$ rad/s. The conductivity of VO_2_ spans several orders of magnitude when it is changed from an insulator state to a metallic state by thermal and electrical stimuli [40]. As VO_2_ is difficult to control during its transition, two stable states of VO_2_ were taken into consideration for investigating the absorber. For the conductivity of VO_2_ ($\sigma_{{\mathrm{VO}}_{2}}$) in Equation (6), we considered values of 10 S/m and $2\times 10^{5}$ S/m for the insulator state and metallic state, respectively [30,41]. A sharp increase in conductivity makes the impedance of an absorber mismatched with the free space impedance of some frequencies, and the absorption can be changed by this transition.

### 2.3. Model Design

**Figure 2:**
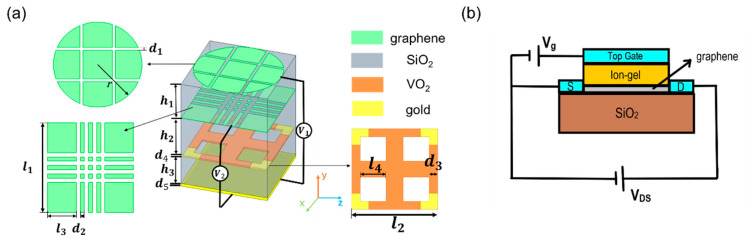
(**a**) A schematic view of the graphene–VO_2_ based absorber and a top view of each layer. (**b**) Instructure of the ion-gel gate device that controls the chemical potential of the graphene. Here, S = source and D = drain.

Figure 2a shows a schematic view of the proposed structure for this paper. The dual-tunable THz absorber is composed of two graphene film layers and one VO_2_-gold based layer, sandwiched between a SiO_2_ layer with a relative permittivity of 2.25 [42]. Each layer can be fabricated by stacking a chemical vapor deposition (CVD)-grown patterned-graphene on the dielectric spacer (SiO_2_) which is supported by a back plate. The thicknesses of the SiO_2_ layers are *h*_1_, *h*_2_, and *h*_3_ from bottom to top, respectively. Here, the dielectric layers act as resonance absorption spaces. The bottom of the structure is a gold mirror with a thickness of *d*_5_ = 0.2 μm. This gold mirror is thick enough to block the propagation of electromagnetic waves and reflect the energy reduced in graphene. Absorption is strengthened by this part of the gold mirror. The first layer is an ultra-thin patterned graphene film, formed by subtracting four rectangular films from a disk with a radius of 2.7 μm. The width of the interval between these different parts is *d*_1_. For the second layer, eight rectangular films with a side length of *l*_1_ and interval width *d*_2_ were subtracted from a square patterned graphene. The third layer consists of a crossed-shaped VO_2_ with four gold parts in the corners of the square. The square has a thickness of *d*_4_, and the side length of the outsider square is *l*_1_ and the side length of the hollow part is *l*_4_. The absorption of the different layers affects each other, which enhances overall absorption and expands bandwidth. The electric field of the patterned graphene design is concentrated to the edge because of the local surface plasmon resonance that contributes to the absorption. Narrow intervals added to the designed pattern can increase the edge of the graphene and can enhance its absorption. Moreover, the whole structure is symmetrical to ensure that the absorber is insensitive to polarizations and incident angles within a large range. The parameters of the absorber proposed are introduced below. Figure 2b displays an ion-gel gate device for tuning the chemical level of the graphene [43]. Additionally, an important factor in the VO_2_’s transition is temperature. It can be tuned using electrifying and terahertz radiation. Adulteration is sometimes used to change the transition temperature in order to suit practical working conditions.

The initial chemical potential of the graphene is assumed as *μ*_c_ = 0.8 eV. VO_2_ is in an insulator state with a conductivity of 10 S/m. Both the finite element method and ANSYS HFSS are applied to analyze the absorption characteristics of the absorber. A THz wave is applied perpendicular to the upper graphene surface. The periodic boundary conditions are used for the x-direction and the y-direction, and the Floquet ports are set in the z-direction. The absorption of the absorber can be calculated as follows:

(7) A(f)=1−R(f)−T(f)

where $R(f)={\left|S_{11}(f)\right|}^{2}$ is the reflection and $T(f)={\left|S_{21}(f)\right|}^{2}$ is the transmission. The *S* parameters are obtained from ANSYS HFSS. Due to the ground gold mirror, the T(f) is zero and the expression of the absorption can be simplified as follows:

(8) A(f)=1−R(f)

Therefore, a relatively high absorption can be obtained when the reflection efficiency is close to zero. Furthermore, the equivalent input impedance ($Z_{\mathrm{in}}$) should match the free space impedance ($Z_{o}$). The size of the patterned graphene and VO_2_–gold layers and the thickness of the dielectric layers can affect the input impedance of the whole structure. To facilitate simulation work, the selection and optimization of the proposed structure parameters were conducted by using the Optimetrics function in ANSYS HFSS. The parameters proposed in Figure 2a are listed in Table 1 for an intuitive view. These parameters make the impedance of the structure match the free space within the high absorption range.

## 3. Results and Discussion

**Figure 3:**
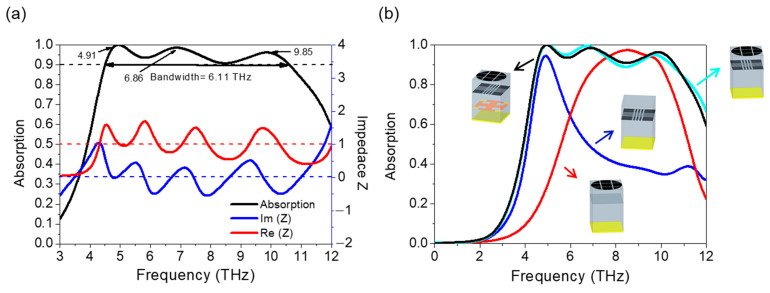
(**a**) The absorption curves and normalized impedances of the absorber when *μ*_c_ = 0.8 eV and VO_2_ is in an insulator state. (**b**) The absorption curves of different parts of the absorber: the black line represents the absorber with three layers; the blue-green line represents the absorber with two top layers; the blue line represents the absorber with the second layer; and the red line represents the absorber with the top layer.

In this section, both the broadband absorption of the proposed structure and the absorptions of different parts of the structure are displayed and analyzed in Figure 3a,b, respectively. The TE incident wave is chosen. Under the simulation conditions set above, the whole structure shows an absorption of over 90% in the frequency range of 4.5–10.61 THz (6.11 THz bandwidth), which is wider than most of the existing broadband THz absorbers proposed. Additionally, the chemical energy of the two graphene layers is set to 0.8 eV, thereby making it more convenient to realize in the experiment. The central frequency (*f*_c_) is 7.56 THz, and the fractional bandwidth is 80.87%. It can be concluded that the real part of the impedance is close to 1 and that the imaginary part is close to 0, which demonstrates that the impedance of the absorber matches the free space. To further discuss the function of multilayered structures in achieving broadband absorption, the absorption curves of both single-layer and multi-layer structures are displayed in Figure 3b. Two graphene layers with different patterns appear to have two distinctive ranges of absorption over 90%. With only one layer of patterned graphene, an absorption of over 90% is relatively narrow. When two layers of patterned graphene act together, the bandwidth has an obvious broadening. The simulated results indicate that a multilayer structure can broaden the absorption range of the absorber.

In order to further disclose the mechanism of the absorber, electric field distributions of the absorber have been plotted. The frequencies corresponding to the absorption peaks of the curves plotted in Figure 3a are listed in Table 2 to make browsing the information more intuitive.

**Figure 4:**
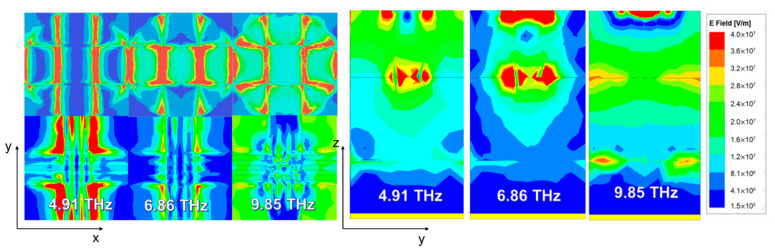
The x-y perspective and y-z perspective of electric field distribution when *μ*_c_ = 0.8 eV and VO_2_ is in an insulator state at absorption peaks of 4.91 THz, 6.86 THz, and 9.85 THz.

In order to further discuss the mechanism of the broadband absorption mode of the absorber, the electric field distributions from both an x-y perspective and y-z perspective are given in Figure 4. In the left of Figure 4, the electric field distribution is observed from an x-y perspective with different frequencies of 4.91 THz, 6.86 THz, and 9.85 THz, respectively. The upper half represents the distribution of the top circle layer, while the lower half represents the electric field distribution in the second graphene layer. In general, we found that the electric field primarily concentrates near the edges of graphene with all resonant frequencies, which indicates that local electric resonance occurs in these areas. Electric dipole resonance is also activated at the edges of the graphene layer [44]. As shown in the top graphene layer, when the simulation resonant frequency increases, edges with the peak value electric field intensity are decreased while other parts of graphene tend to have an increase in the electric field intensity. The electric field intensity becomes more dispersed and covers the entire graphene, which can be explained as a result of the local surface plasmon resonance of graphene being excited by the incident THz wave [45]. In the second graphene layer, the electric field distribution has a trend of decreasing first and then increasing with increasing simulated frequency. Similar to the results in the top layer, the peak value of the electric field at the edge of graphene decreases and an increase occurs in other parts of the graphene layer. The incident wave excites carriers and induces tangential electric fields on both the top graphene layer and the second graphene layer.

The electric field intensity distributions in the y-z plane of the absorber at each peak frequency are plotted in the right side of Figure 4. By comparing the electric field intensity of different parts, the important part for incident wave absorption at each resonant frequency can be distinguished. In principle, the energy consumption and optical loss in materials can be represented by the following equation:

(9) $$A(\lambda )=2\pi \frac{c}{\lambda }\varepsilon^{"}{\int_{V}\left|E_{l}\right|}^{2}dV$$

where $\varepsilon^{\prime \prime }$ is the imaginary part of the dielectric permittivity, V is the volume of the material, V is the electric field inside the material, and *c* is the speed of light in a vacuum. The electric field is trapped in graphene layers, in the VO_2_ layer, and in the SiO_2_ dielectric layers. However, the absorption effect of the dielectric layers is less effective than other parts, corresponding to electric distribution. This can be explained by Equation (9); the imaginary parts of graphene and VO_2_ permittivity are larger than the dielectric layers within the THz range. In resonant frequencies of 4.91 THz and 6.86 THz, energy consumption mainly occurs in the top and second graphene layers. As the resonant frequency rises to 9.85 THz, the energy consumption of the VO_2_ layer is strengthened since the electric field intensity increased.

**Figure 5:**
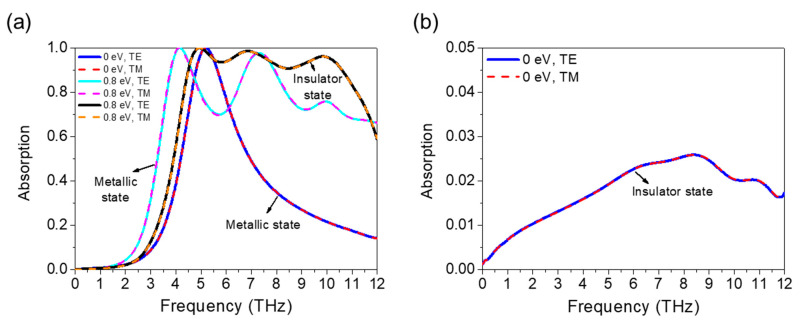
(**a**) Broadband absorption of an absorber with 0.8 eV Fermi energy levels and insulator VO_2_ states, dual-band absorption with 0.8 eV Fermi energy and metallic VO_2_ state, and single-band absorption with 0 eV and metallic absorption (TE and TM polarization). (**b**) Absorption with the Fermi energy level 0 eV and VO_2_ insulator state (TE and TM polarization).

The switchable function of the THz absorber is achieved by tuning both the Fermi energy level and the phase transition property of the VO_2_. The structure acts as a broadband THz absorber when *μ*_c_ is 0.8 eV and VO_2_ is in an insulator state and as a dual-band absorber when *μ*_c_ is 0.8 eV and VO_2_ is in a metallic state. Additionally, when the voltage applied to the graphene layers is set to 0, the device acts as a single-band absorber when the VO_2_ is in a metallic state and as a broadband reflector when the VO_2_ is in an insulator state. The reflectivity is greater than 97% because the transmission coefficient of the absorber is 0 and the absorption is less than 0.03. As shown in Figure 5a, the dual-band absorption has absorption peaks of 4.16 THz and 7.3 THz and the single-band absorption has an absorption band of 4.81–5.65 (0.84 THz). The simulated results show that the switchable working characteristics of the structure can be controlled and changed significantly.

**Figure 6:**
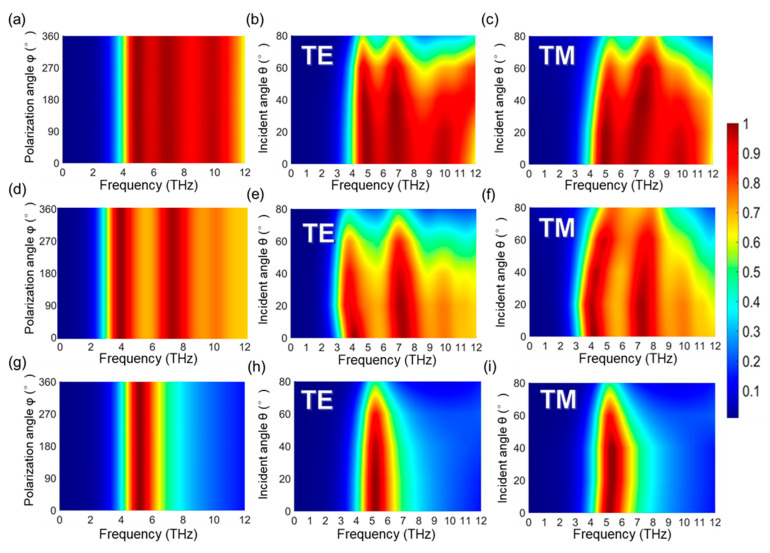
Absorption spectra with different polarization angles (*φ*) and incident angles (*θ*) and frequency of the incident wave from 0 to 12 THz: (**a**–**c**) broadband absorption when *μ*_c_ = 0.8 eV and VO_2_ is in an insulator state; (**d**–**f**) dual-band absorption when *μ*_c_ = 0.8 eV and VO_2_ is in a metallic state; (**g**–**i**) single-band and relatively narrow absorption when *μ*_c_ = 0 eV and VO_2_ is in a metallic state.

It is of great importance to design absorbers equipped with polarization insensitivity and tolerance within a wide incident angle (*θ*) range. In this section, absorptions of the proposed absorber are plotted in contour maps to analyze their insensitivities in both the incident angles and polarization angles. In Figure 6, three switchable absorption modes of the absorber (broadband absorber, dual-band absorber, and single- and narrow-band absorber) are discussed with respect to different polarization angles *φ* and incident angles *θ*. In Figure 6a,d,g, the absorption spectra of the broadband, dual-band, and single narrow band are shown with different polarization *φ*. From our results, it can be concluded that the absorber is polarization-insensitive. This can be ascribed to the asymmetrical design of the structure. For broadband absorption, spectra with TE and TM polarized waves are represented in Figure 6b,c, respectively. Three absorption peaks in this absorption mode can keep the absorption over 80% up to a 55° incident angle for the TE mode and up to a 60° incident angle for the TM mode. As shown in Figure 6e,f, for the TE polarized incident wave, the two absorption peaks of the dual-band absorber can keep absorption over 80% up to an incident angle of 60°. The dual-band absorber can maintain stable absorption up to 65° for the TM mode, and the absorption peaks tend to have a blue shift with an increasing incident angle. In Figure 6h,i, the absorption spectra of relatively narrow and single-band absorbers with TE and TM modes are displayed, respectively. Both of these absorbers can keep the absorption peak over 80% up to an incident angle of 65°. Great tolerance for incident angle is obtained by the subwavelength structure, which can facilitate the match between the structure and the wave from free space. Overall, the switchable THz absorber proposed in this work possesses an incident angle insensitivity and a polarization insensitivity in different absorption modes.

**Figure 7:**
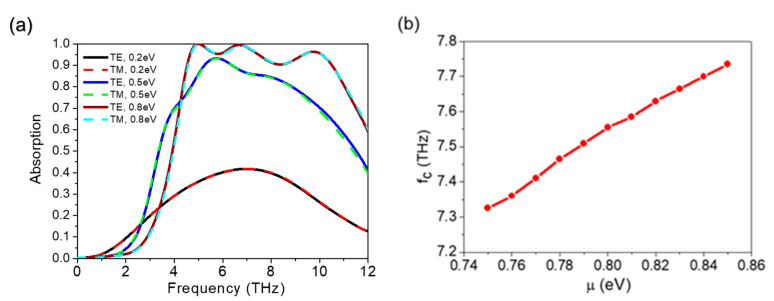
(**a**) Absorptions with different polarizations; Fermi energy levels (*μ*_c_) of 0.2 eV, 0.5 eV, and 0.8 eV; and $\tau =\frac{\mu \mu_{c}}{ev_{F}^{2}}$. (**b**) Central frequencies with consecutive Fermi energy levels from 0.75 eV to 0.85 eV.

Finally, we discuss the tunable function for the broadband absorber of Fermi energy level of the graphene and the relaxation time τ. In practical situations, different energy levels (*μ*_c_) for two graphene layers are hard to implement. Therefore, the Fermi energy was set the same for each graphene layer. In Figure 7a, the values of *μ*_c_ are set to 0.2 eV, 0.5 eV, and 0.8 eV, respectively. Because the relaxation time is in direct proportion to the chemical potential *μ*_c_, the following expression is given: $\tau =\frac{\mu \mu_{c}}{ev_{F}^{2}}\;$. Here, *μ* is the electron mobility decided by the quality of the graphene and is fixed on 1250 cm^2^/Vs. Therefore, the relaxation time in Figure 7a is 0.025 ps, 0.0625 ps, and 0.1 ps for 0.2 eV, 0.5 eV, and 0.8 eV, respectively. Here, the VO_2_ is in an insulator state. The absorption curves of both TE and TM polarized waves are then calculated. The results indicate that the structure proposed can support different polarized incident waves. As *μ*_c_ increases, the absorption increases gradually and absorption peaks start to appear. Additionally, the quantity of the absorption peaks increases and the broadband absorption appears. The relationship between the Fermi energy *μ*_c_ and the conductivity of graphene is introduced in Section 2.1. The higher the value of *μ*_c_, the greater the surface impedance of the graphene. This change leads to an increase in absorption. Moreover, the relationship between central frequency (*f*_c_) and consecutive Fermi energy levels varies within a range of 0.75 eV–0.85 eV. Tuning μ_c_ in the range of 0.8 eV ± 0.05 eV, the *f*_c_ of the broadband absorber can be tuned linearly. It can be concluded that the graphene also plays an important role in controlling the structure. The relaxation time (*τ*) of the patterned graphene is investigated because it can be changed by the quality of the graphene [46].

In order to compare our absorber with other absorbers in the references, we listed the main properties of broadband absorbers in Table 3. Compared with other structures, the hybrid absorber exhibits both a significantly larger bandwidth and a higher fractional bandwidth with a small number of layers, showing great potential for miniaturized broadband THz applications.

## 4. Conclusions

In summary, a switchable, dual-tunable THz absorber based on patterned graphene and phase-change VO_2_ was proposed and investigated. When VO_2_ was in an insulator state, the device could be switched from near-total reflection (>97%) to high absorption over a wide frequency range (4.5–10.61 THz) as the Fermi level of graphene changed from 0 to 0.8 eV. When VO_2_ was transmitted to a metal state, the device acted as a single-band absorber with absorption band 0.84 THz (when the Fermi energy level of graphene is 0 eV). When VO_2_ was in a metal state and the Fermi energy level of graphene was tuned to 0.8 eV, the device acted as a dual-band absorber with bands of 3.78–4.61 THz and 6.75–7.94 THz. The absorber was polarization-insensitive and could maintain a stable high absorption performance within a 55° incidence angle. Moreover, the broadband structure could maintain a stable absorption within a *τ* change of ±50%. With respect to the large absorption bandwidth, flexible tunability, and switchable functionality, the device is promising for various terahertz applications.

## Figures and Tables

**Table 1:** Parameters of the proposed structure (μm).

Parameter	Size	Parameters	Size	Parameter	Size
*d*_1_	0.18	*d*_5_	0.2	*l*_1_	1.05
*d*_2_	0.26	*l*_1_	5.4	*h*_1_	2.15
*d*_3_	0.5	*l*_2_	5.25	*h*_2_	2
*d*_4_	0.5	*l*_3_	1.8	*h*_3_	2.5

**Table 2:** Absorption peaks in broadband absorption.

*μ*_c_ and VO_2_ State	Absorption Peaks and Corresponding Frequencies					
0.8 eV Insulator	Peak I		Peak II		Peak III	
	4.91 THz	100%	6.86 THz	98.50%	9.85 THz	96.35%

**Table 3:** Comparison of important properties of THz absorbers.

References	Absorption Bandwidth (THz) ^1^	Material	Layers	Stable Incident Angle	Polarization Insensitivity
[19]	2.76 (2.14–4.90)	Graphene	1	Up to 60°	Insensitive
[11]	3.7 (1.95–5.65)	Gold	1	Up to 20°	Insensitive
[1]	0.76 (4.80–5.56)	Graphene	3	Up to 60°	Insensitive
[18]	2.2 (0.6–2.8)	Graphene	3	Up to 60°	Insensitive
[22]	3.5 (6.98–9.1)	Graphene	3	Up to 60°	Insensitive
This work	6.11 (4.5–10.61)	Graphene and VO_2_ (0.8 eV insulator state)	3	TE up to 55° TM up to 60°	Insensitive
	0.83 (3.78–4.61) 1.19 (6.75–7.94)	Graphene and VO_2_ (0.8 eV metallic state)	3	TE up to 60° TM up to 65°	Insensitive
	0.84 (4.81–5.65)	Graphene and VO_2_ (0 eV metallic state)	3	TE up to 65° TM up to 65°	Insensitive

^1^ Bandwidth of absorption over 90%.
